# Malpighian tubules are important determinants of *Pseudomonas* transstadial transmission and longtime persistence in *Anopheles stephensi*

**DOI:** 10.1186/s13071-015-0635-6

**Published:** 2015-01-21

**Authors:** Ali Reza Chavshin, Mohammad Ali Oshaghi, Hasan Vatandoost, Bagher Yakhchali, Fahimeh Zarenejad, Olle Terenius

**Affiliations:** Social Determinants of Health, Research Center, Urmia University of Medical Sciences (UMSU), Urmia, Iran; Department of Medical Entomology and Vector Control, School of Public Health, Urmia University of Medical Sciences (UMSU), Urmia, Iran; Department of Medical Entomology and Vector Control, School of Public Health, Tehran University of Medical Sciences (TUMS), Tehran, Iran; Institute for Environmental Research (IER), Tehran, Iran; Department of Environmental Biotechnology, National Institute of Genetic Engineering and Biotechnology (NIGEB), Tehran, Iran; Department of Ecology, Swedish University of Agricultural Sciences (SLU), Uppsala, Sweden

**Keywords:** Malaria, *Anopheles stephensi*, *Pseudomonas*, GFP, Transstadial transmission, Paratransgenesis

## Abstract

**Background:**

*Pseudomonas* is a genus of bacteria commonly found in investigations of gut microbes in malaria mosquitoes. Among those mosquitoes is the dominating malaria vector in Asia, *Anopheles stephensi*, where *Pseudomonas* is a prevailing bacterium and natural inhabitant of its breeding places. In order to explore the reason for finding *Pseudomonas* so frequently, an investigation of its localization and transstadial properties was undertaken.

**Methods:**

A *Pseudomonas* isolate from *An. stephensi* was transformed successfully with an endogenous plasmid modified to express green fluorescent protein (GFP). Subsequently, the *Pseudomonas*-GFP was added to the laboratory larval breeding place of *An. stephensi* and taken up by the larvae. After 24 hours, the larvae were cleaned and moved to a bath with double-distilled water. Also, female adults were fed sugar solution containing *Pseudomonas*-GFP. The *Pseudomonas*-GFP was traced in the alimentary canal of larvae, pupae and adults.

**Results:**

Fluorescent microscopy and PCR assays showed that the *Pseudomonas* bacteria underwent transstadial transmission from larvae to pupae and then to adults. In blood-fed female mosquitoes, the bacteria increased in numbers and remained in the mosquito body for at least three weeks after eclosion. In addition to the midgut, the Malpighian tubules of both larvae and adult mosquitoes were colonized by the bacteria. Also *Pseudomonas*-GFP that was distributed through sugar solution was able to colonize the Malpighian tubules of adult females.

**Conclusions:**

Colonization of the Malpighian tubules by *Pseudomonas* bacteria seems to be important for the transstadial passage from larvae to adult and presumably for the longevity of the bacteria in the adult mosquito. The existence of an entry point in the larval stage, and the long duration in the female gut, opens up for a possible use of *Pseudomonas* in mosquito paratransgenesis.

## Background

Insect gut bacteria live in an environment where the host organism and other microorganisms influence the conditions. One can expect an element of coadaptation between the bacteria and their host at least for bacteria that are beneficial for the host for food degradation or for suppression of harmful bacteria. Some bacteria are living in such close relationship with their insect hosts that they now lack certain functions that would enable them to live outside their host. Well-studied examples include the microbiota in termites [1] and the genus *Buchnera* in aphids [2]. Also some important vectors of disease have obligate midgut bacterial symbionts. For example, the vector for Chagas disease, the kissing bug *Rhodnius prolixus* that carry blood-degrading *Rhodococcus rhodnii* [3] and tsetse flies that carry the symbiont *Wigglesworthia glossinidia* [4]. In mosquitoes, two of the key questions regarding gut bacteria have concerned persistence of bacteria and whether bacteria can be transferred over developmental stages, so called transstadial transmission. For certain bacteria, such as *Wolbachia*, recently found in *Anopheles gambiae* and *Anopheles coluzzi* [5], the intracellular life of the bacteria secure a transstadial transmission. Likewise, the genus *Asaia* has been shown to infect reproductive tissues and also undergo transstadial transmission [6,7].

Mosquito-borne diseases are serious health problems around the world and more than half of the world population is at risk of getting infected. For several mosquito-borne diseases, especially in the case of malaria, control mainly depends on the use of insecticides and related methods such as insecticide-treated nets (ITNs). However, while great achievements have been made using routine malaria control programs, the current tools are at stake because of problems arising such as insecticide resistance in *Anopheles* mosquitoes, drug resistance in the malaria parasites and lack of an effective vaccine. Consequently, this has forced researchers to develop other methods including transgenesis and paratransgenesis. For vector transgenesis, the goal is to genetically modify vectors [8], while for paratransgenesis, the goal is to modify symbiotic bacteria of the vector to deliver anti-parasitic effector molecules to wild vector populations. Paratransgenesis can also be used to express substances with pathogenic effects that interfere with the mosquito reproduction and shorten the life span [6,8,9]. The basic and most important step in paratransgenesis is to find suitable bacteria. They should dominate the insect microflora, infest a part of vector body where they are in close contact with the pathogen, and be adapted to the immunological and physiological condition of the vector body. Also, transstadial transmission is an important criterion since it would enable the genetically engineered bacteria to be introduced into the larval breeding places and then be naturally transferred to the adults.

*Pseudomonas* bacteria have in previous studies often been a common member of the gut flora in malaria mosquitoes (Table 1). From the studies performed, it seems that *Pseudomonas* is more abundant in *Anopheles* mosquitoes from the Indian subcontinent and the Arabian Peninsula than in Africa, and also more abundant in *An. stephensi* than in other species. Whether this is a general fact and whether this is dependent on mosquito species or environmental differences remains to be seen. Recently, it was shown that in *Anopheles culicifacies* and *An. stephensi* identical sequences of 16S for *Pseudomonas* bacteria were found in both larvae and adults indicating that a transstadial transmission was possible [10,11]. In this study, transstadial transmission, colonization site, and the effect of blood- and sugar-feeding was investigated for a *Pseudomonas* isolate from *Anopheles stephensi*, the main malaria vector in Asia.

**Table 1:** **Presence of** ***Pseudomonas*** **in studies on** ***Anopheles*** **gut flora**

**Mosquito species**	**Frequency**	**Reference**	**Region**
*An. albimanus*	17%	[12]	Laboratory reared, USA, North America
*An. culicifacies*	Most common, 57%	[10]	Iran, Asia
*An. darlingi*	5% (3 of 57)	[13]	Brazil, South America
*An. gambiae*	17%	[12]	Laboratory reared, USA, North America
*An. gambiae*	1.65%	[14]	Cameroon, Africa
*An. gambiae*	0.05-5.37% depending on stage	[15]	Partly laboratory reared, Kenya, Africa
*An. maculipennis*	*Pseudomonas* sp. were detected in most of the specimens analyzed	[16]	Iran, Asia
*An. stephensi*	38%	[12]	Laboratory reared, USA, North America
*An. stephensi*	*Pseudomonas* sp. were detected in most of the specimens analyzed	[16]	Iran, Asia
*An. stephensi*	*Pseudomonas* was the most frequent (51% in larvae and 54% in adults)	[11]	Iran, Asia
*An. stephensi*	*Pseudomonas mendocina* and *Serratia marcescens* were the most abundant	[17]	Laboratory reared, India, Asia

## Methods

### Bacterial transformation

A *Pseudomonas* species was previously isolated from *An. stephensi* larvae [11] and selected for this study because of its dominant presence both in larvae and adults. The isolate (BND-YL1; GenBank accession no. HQ832847) is most closely related to *Pseudomonas extremaustralis*. A ~ 4 kb endogenous plasmid was successfully extracted from the BND-YL1 isolate using a high pure plasmid isolation kit (Roche) according to the manufacturer’s instructions. After evaluating several enzymes, the extracted plasmid was digested with *Bam*HI and *Hin*dIII and a GFP gene was inserted using T4 DNA ligase. Competent cells of the BND-YL1 isolate were transformed by electroporation [18] with its native plasmid expressing GFP. The transformed bacteria were examined by fluorescent microscopy and PCR amplification with GFP-specific primers. Colonies with successful GFP expression (hereafter called *Pseudomonas-*GFP) were selected and used for further studies.

### Establishment of *Pseudomonas*-GFP in mosquitoes

For experiments we used a laboratory-reared strain of *An. stephensi* (originally from south of Iran and kept at Department of Medical Entomology, School of Public Health, Tehran University of Medical Sciences, SPH-TUMS). The mosquitoes were reared at 25 ± 2°C with 70% humidity.

Fifty first instar larvae were reared in trays including 10^6^ CFU *Pseudomonas*-GFP plus ampicillin 100 μg/ml. After 24 hours, the larvae were removed and washed twice in separate baths of ddH_2_O to eliminate the bacteria on the larval body surface and then transferred to ddH_2_O plus ampicillin (100 μg/ml). The larvae were reared for the rest of their lives in the ampicillin-added ddH_2_O. The larvae were left to pupate, and emerged at adult stage from the same water they pupated in. At least two individuals were dissected from each stage (L2, L3, L4 and pupae). The presence of GFP-producing bacteria in their organs was examined by microscopy.

### Adult experiments using *Pseudomonas-*GFP

Two parallel experiments were performed for adults. In the first experiment, the larvae which acquired *Pseudomonas-*GFP bacteria were allowed to pupate and eclose. The emerged female adults were dissected two, three, and ten days after eclosion (Table 2). The females were artificially blood fed with defibrinated cow blood on day three and on day ten in order to study the effect of blood meals on colonization of *Pseudomonas*-GFP in mosquito midguts and other organs. The blood fed mosquitoes were dissected and examined by fluorescent microscopy 24, 48 or 72 h post blood feeding (Table 2).

**Table 2:** **Dissection scheme with number of adult mosquitoes dissected per time point** ^**a**^

**Hours post blood meal**	**Days after emergence**		
	**2**	**3**	**10**
0^b^	3	3	3
24		3	3
48		3	3
72		3	3

^a^This scheme applies for each of the groups of mosquitoes fed with *Pseudomonas*-GFP as larvae, adults fed with sugar containing *Pseudomonas*-GFP, and the controls of respective experiment.

^b^Before blood feeding.

In the second experiment, female adults raised without *Pseudomonas*-GFP were fed with sugar containing *Pseudomonas*-GFP bacteria. These adults were then blood fed and examined as above.

### Detection of GFP-expressing *Pseudomonas*

The mosquitoes were examined by fluorescent microscopy using a BX61 microscope, with U-LH100HGAPO lamp houses and DP-70 imaging system (Olympus). In all fluorescent microscopy surveys, larvae or adult mosquitoes with no exposure to the GFP-expressing bacteria were examined as negative controls to ensure that the green light observed originated from the GFP-expressing bacteria and not was due to auto-fluorescence.

To reconfirm the presence of *Pseudomonas*-GFP bacteria in the mosquitoes we performed PCR with GFP-specific primers. Briefly, before dissection the mosquito surface was sterilized with 70% ethanol [19]. Then the specimen was dissected under sterile conditions, and the midgut was mashed and suspended in 500 μL of Brain Heart Infusion (BHI) and incubated for 24–48 hours at 37°C. DNA of the grown bacteria was extracted using QIAGEN DNeasy Kit (Qiagen, Germany) according to the manufacturer′s instructions. The GFP-gene primers, GFP-F 5΄-CAAGAGTGCCATGCCCGAAGG-3΄ and GFP-R 5΄-GACAGGGCCATCGCCAATTGG-3΄, were used to obtain a 280 bp band [20,21]. The PCR conditions were 94°C for 10 minutes, followed by 35 cycles of [95°C for 30 seconds, 62°C for 40 seconds, 72°C for 30 seconds], and 72°C for 8 minutes.

## Results and discussion

In a number of studies, larval midgut bacteria has been said to be transmitted transstadially to the adult gut [12,22-24]. In most of these studies, the same bacteria have been found both in larvae and in adults, which could be interpreted as transstadial transmission. These findings contradict the common notion that the midgut bacteria of mosquito larvae are removed during metamorphosis and formation of the peritrophic membrane in larvae [25]. An alternative explanation to finding the same bacteria in the adults as in the larvae, could be that immediately after emergence adult mosquitoes take a sip of water from their hatching environment [26]. However, in *An. stephensi* we have shown that for *Escherichia coli* the bacterial persistence could also be due to escape from the metamorphosis using the Malpighian tubules as a covert [27]. It is known that the larval Malpighian tubules remain intact during metamorphosis in *Drosophila* [28,29] and tubular fluid including the bacteria may drain from Malpighian tubules towards the midgut at the junction point of midgut/hindgut and Malpighian tubules. To further explore the possibility of a Malpighian-tubules hide-out as an explanation for the common presence of *Pseudomonas* in malaria mosquitoes, we investigated whether *Pseudomonas* belonging to the natural flora of *An. stephensi* [11], also are able to colonize the Malpighian tubules in a similar manner.

**Figure 1:**
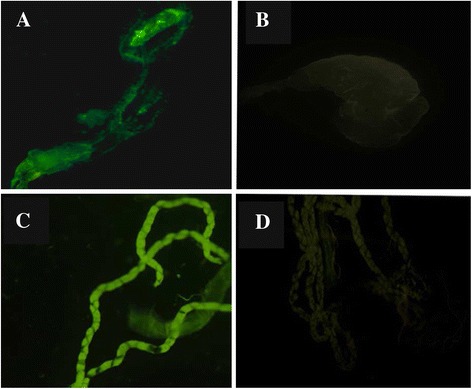
**Colonization of** ***Pseudomonas*** **-GFP in larval midgut and Malpighian tubules. (A)** midgut, **(B)** midgut negative control **(C)** Malpighian tubules, **(D)** Malpighian tubules negative control.

**Figure 2:**
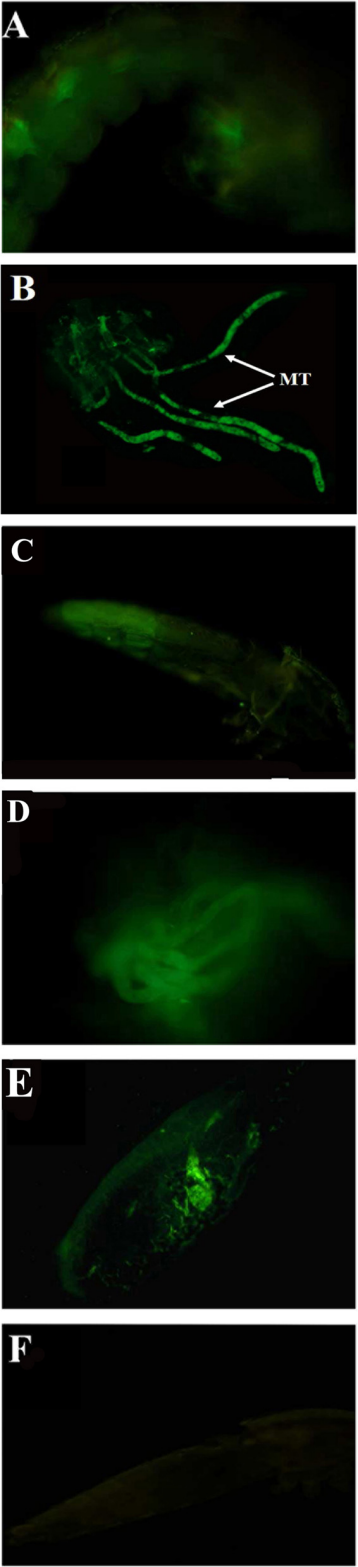
**Colonization and transstadial transmission of** ***Pseudomonas*** **-GFP bacteria in** ***An. stephensi*** **. (A)** live pupa, **(B)** Malpighian tubules of dissected pupa, **(C)** live adult, **(D)** Malpighian tubules of live adult, **(E)** dissected adult midgut, **(F)** live adult negative control.

We initiated the investigation by successfully transforming an endogenous plasmid into the *Pseudomonas* isolate BND-YL1, as determined by GFP expression and PCR amplification of the GFP gene sequence (data not shown). Then the genetically transformed *Pseudomonas* was added to the breeding water of 1^st^ instar *An. stephensi* larvae. After 24 hours, the larvae were cleaned and moved to a bath with ddH_2_O. At the 4^th^ instar stage, all larvae showed expression of GFP in the larval midgut (Figure 1A), but the colonization was particularly intense in the Malpighian tubules (Figure 1C). GFP expression was also seen in all pupae and adults (Figure 2). The transstadial transmission of the *Pseudomonas* isolate BND-YL1 was confirmed both by fluorescent microscopy and PCR amplification of the GFP gene.

**Figure 3:**
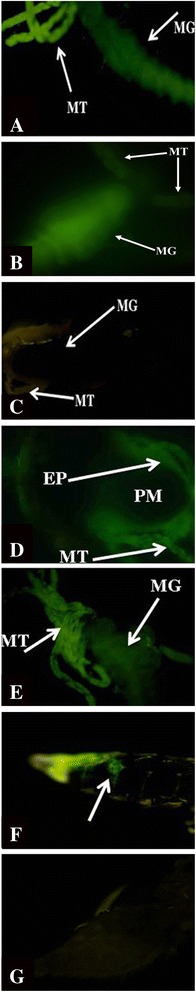
**Colonization of** ***Pseudomonas*** **-GFP in adult females. (A-C)** Dissected mosquitoes after feeding sugar containing *Pseudomonas-*GFP; **(A)** One week old, no blood feeding; **(B)** 3 weeks old, blood-fed at days 3 and 10; **(C)** negative control; **(D-G)** Mosquitoes receiving *Pseudomonas-*GFP as larvae, 48 hours after third gonotrophic cycle; **(D-E)** Dissected mosquitoes; **(F)** Abdomen of live mosquito; **(G)** Negative control. (EP) epithelial cell line. (MG) midgut. (MT) Malpighian tubules. (PM) peritrophic matrix.

The successful transstadial transmission opened up for exploring the potential use of *Pseudomonas* for mosquito paratransgenesis; a pathogen-interference approach in which a genetically modified bacterium produces substances incapacitating the pathogens in adult mosquitoes [30]. Introducing a genetically modified symbiont into wild populations is regarded as more feasible in the larval stage where mosquito larvae are concentrated in breeding sites and also largely feed on bacteria. Therefore our discovery of bacteria having the ability of transstadial transmission could be an important step in a paratransgenesis approach for malaria control. An alternative approach is to supply adult females with modified bacteria in sugar containers [30]. Therefore, we also investigated whether the bacteria could reach the Malpighian tubules by feeding sugar solution containing *Pseudomonas*-GFP. Indeed, also all 27 female adults revealed successful bacterial establishment in the mosquito Malpighian tubules (Figure 3).

However, to interact with malaria parasites, the bacteria must also proliferate in the gut upon a blood meal and preferably remain in the gut a prolonged time to be established at renewed blood meals. To explore the effect of blood feeding on the *Pseudomonas* bacteria, female *An. stephensi* were offered blood. The number of *Pseudomonas* bacteria increased substantially after a blood meal, which is in agreement with previous studies [27,31-33] that also showed an increased number of gut bacteria post-blood feeding. In *An. gambiae* it was shown that *Pseudomonas* bacteria had a statistically significant increase after a blood meal and that it could be linked to its ability to withstand oxidative stress [15]. The fluorescent intensity was highest about 24–48 hours after blood feeding (Figure 3D-F) and in our experiments, where the mosquitoes were blood fed once a week, fluorescent bacteria could be observed in the mosquito midgut more than 3 weeks (Figure 3B). Each gonotrophic cycle takes about two-three days under our lab conditions; therefore, the bacteria remained alive without additional blood meal nutrients for at least four to five days more. The 3 week-survival of *Pseudomonas* in the mosquito body is enough to cover a period of several potential *Plasmodium* infections in the vector body and is a substantial increase in time as compared to our previous data with GFP-expressing *E. coli* that remained 13 days in mosquitoes of the same colony [27].

The colonization of Malpighian tubules could also make *Pseudomonas* species potential candidates for paratransgenesis against pathogens for which a part of the life cycle is related to Malpighian tubules. For instance, the method could be used against *Dirofilaria immitis*, one of the causing agents of zoonotic dirofilariasis, which is endemic in several parts of southern Europe [34], United States of America [35], some parts of Asia [36], and the Middle East [37]. One has to consider though that among the more than 100 *Pseudomonas* species some are pathogenic. One example is *Pseudomonas aeruginosa*, which is an opportunistic human pathogen in severely immunocompromised patients [38]. Therefore, only *Pseudomonas* species that are non-pathogenic should be selected for a paratransgenesis approach.

## Conclusions

In this study, we showed that *Pseudomonas* bacteria can survive and stay successfully in the midgut of mosquitoes during several molting or ecdysis events during larval stages as well as hydrolytic processes during metamorphosis and finally transfer to adults. Also, the bacteria were localized in midgut as well as Malpighian tubules, increased following blood meals, and remained in the lumen of adult mosquitoes more than 21 days after eclosion. Although our data could suggest that a non-pathogenic *Pseudomonas* bacteria would be useful in a paratransgenesis approach, further studies would be needed to engineer the *Pseudomonas* isolate with some effector molecules and to test the ability of the recombinant bacterium to block parasite transmission in the laboratory and under field conditions.
